# The Life Cycle of Health Technologies. Challenges and Ways Forward

**DOI:** 10.3389/fphar.2017.00014

**Published:** 2017-01-24

**Authors:** Iñaki Gutiérrez-Ibarluzea, Marco Chiumente, Hans-Peter Dauben

**Affiliations:** ^1^Osteba, Basque Office for Health Technology Assessment (HTA), Ministry for Health, Basque GovernmentVitoria-Gasteiz, Spain; ^2^Societá Italiana di Farmacia Clinica e Terapia (SIFaCT) – Italian Society of Clinical Pharmacy and TherapeuticsMilan, Italy; ^3^German Agency for Health Technology Assessment (DAHTA), Deutsches Institut für Medizinische Dokumentation und Information (DIMDI)Cologne, Germany

**Keywords:** disinvestment, life cycle assessment, health technology assessment, investment, innovation, reimbursement mechanisms, real world data

Health care systems have considered the introduction of health technologies a linear process in which different stakeholders (innovators, manufacturers, regulators, health technology assessors, reimbursement bodies, health care providers, health care professionals, patients, and citizens) did interact in each of the steps of the process, but were not involved in a continuous dialogue and knowledge exchange. This step by step approach generates inefficiency in many cases by means of: the isolation of innovators from real health care needs, the introduction of health technologies of doubtful value, the generation of unnecessary variability in practice, the maintenance of practices of no-added value, and the disregard of knowledge out of the practice, among others. These circumstances suppose an inefficient allocation of resources and investment, a hole in the waterline of health care systems, and their sustainability and what is more, a non-direct correlation between expected or theoretical outcomes and real health outcomes. Different initiatives have been put in place in the last years in order to mitigate the effects of the aforementioned issues.

On the basis of the life cycle concept of health technologies (see Figure 1), this article will go through some of these initiatives and define the role that Health Technology Assessment could play in each step.

**Figure 1:**
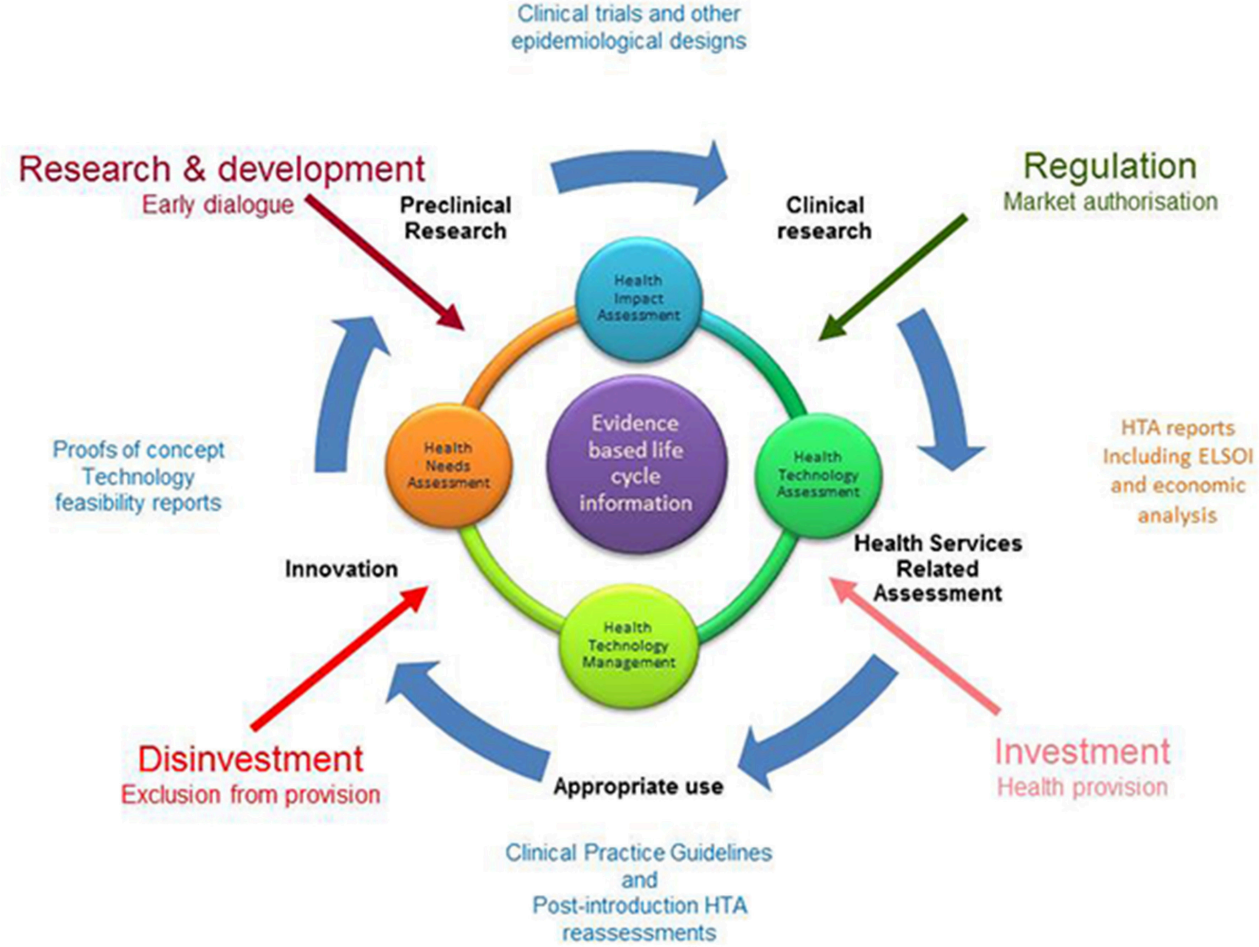
**The life cycle of health technologies concept**. ELSOI (Ethical, Legal, Social and Organizational Issues).

Health Technology Assessment (HTA) is “*the systematic evaluation of the properties and effects of a health technology, addressing the direct and intended effects of this technology, as well as its indirect and unintended consequences, and aimed mainly at informing decision making regarding health technologies. HTA is conducted by interdisciplinary groups that use explicit analytical frameworks drawing on a variety of methods*” HTAglossary (http://htaglossary.net).

HTA was used to act when decisions on reimbursement were required and thus was called the fourth hurdle or the fourth guarantee (safety, efficacy, effectiveness, and efficiency). However, having in mind its definition and privileged position, HTA activities have evolved to more constructive approaches from health technology inception to its obsolescence (Henshall et al., 2011). New concepts such as scientific advice (Jost et al., 2015), early dialogue, early awareness, and alert systems (Packer et al., 2012), post-introduction observation of health technologies (Varela-Lema et al., 2012), appropriateness, re-assessment, and disinvestment (Elshaug et al., 2007) have gained ground.

## Research and development

In the nineties of the 20th century, many countries in the world started policies on incentivizing the creation of innovation hubs and enterprises incubators in different sectors, especially energy, transport, and biosciences-health. These policies were followed by supporting innovators, universities, spin-offs, spin-outs, and small and medium enterprises (SMEs) both managerially and economically. These policies generated an increasing amount of initiatives and technological ideas. One of the challenges of those innovators were centered in convincing the health care systems on the added-value of their ideas and anticipating financial needs, especially regulators, and reimbursement requirements of evidence generation. Regulatory agencies including the European Medicines Agency (EMA) established focused policies to promote innovative solutions and especially the development of new drugs by SMEs. Furthermore, EMA launched in 2005 an “SME Office” to provide financial and administrative assistance to micro-SMEs (Carr, 2010).

Unfortunately, many of these initiatives failed, not linked to their possible value to health systems, but rather due to inadequate planning of the steps to be followed in the continuum from regulation to market access. This misalignment between innovators, regulators, and health care systems have generated that many laboratory discoveries have gone to the termed “valley of death,” the so-called gap between bench research to health care application (Roberts et al., 2012). Moreover, as Roberts et al. (2012) described there are fewer scientists with a true understanding of clinical problems and health needs. The promotion of clinician-scientists is crucial to bridge the gap and avoid the valley of death at the early stages, as well as the contribution of “early dialogue” and scientific advice to define the characteristics of the patient and health care system to which the new technological solution will be targeted to. In fact, some highly sophisticated technological solutions such as many “point of care” diagnostics have not been successful due to an inadequate plan including the inexistence of companion treatments or the insufficient knowledge of the standard of care in different settings. However, there are examples of initiatives that have combined experts, health care providers, and HTA organizations and have become a reality such as the European Union (EU) funded projects AngeLab for non-invasive prenatal diagnosis (http://angelab-systems.eu/) or Discognosis, a Point-of-Care diagnostic device for malaria and other tropical diseases (http://www.discognosis.eu/).

Current initiatives to promote dialogue and funding at the early stages include the Innovative Medicines Initiative (IMI) that aims to facilitate and speed the access to the market and obviously, to the patients of innovative medicines, with an especial focus in areas where there is high unmet medical (e.g., rare diseases) or social need (https://www.imi.europa.eu/). IMI is a public-private joint action with the participation of the European Union (through the European Commission) and the European pharmaceutical industry represented by the European Federation of Pharmaceutical Industries and Associations (EFPIA).

## Regulation

Market authorization or regulation has been a necessary step that countries and health care systems have established to build trust on the products that are marketed authorized, in terms of safety and efficacy/performance. Developed countries have created well-structured and demarcated processes for market authorization, especially when they relate to health and environment. These necessary steps have been followed by the creation of independent institutions at the national (Food and Drug Administration and other national bodies) and international level (EMA) that have defined the rules and evidence requirements to ensure that the products that reach the market are safe and efficacious. Nevertheless, these regulatory processes and evidence requirements for market access are unequal for the different health technologies. We need to bear in mind that health technology definition embraces “*any intervention developed to prevent, diagnose or treat medical conditions; promote health; provide rehabilitation; or organize healthcare delivery. The intervention can be a test, device, medicine, vaccine, procedure, program or system*” (http://htaglossary.net/health+technology). Meanwhile, drug development and market access is well-defined and regulated in many countries in the world (at least in OECD countries), medical devices, diagnostics, procedures, programs, or systems do not currently follow such process. In fact, researchers and health technology assessors have claimed for a more robust and centralized system for medical devices in Europe (Eikermann et al., 2013) that could enhance not only the evidence generation necessary for decision making, but the competitiveness of European medical devices when accessing other markets. Another debate could be the possible evidence requirements of procedures and programs to ensure that only those that comply with high-quality standards are promoted to health care systems. An open area under discussion is public health interventions. Also here a higher level of evidence is demanded but due to the nature of the interventions, the tools and knowledge on how to handle evidence quality is lacking.

It is also worth noting that health care systems, services providers, and health technologies manufacturers are demanding an alignment of evidence requirements by regulators and reimbursement bodies. In this sense, regulators, industry, and HTA bodies have started common approaches to outcomes of interest definition, stakeholders' involvement, and possible comparators when designing trials for market authorization of drugs and medical devices. A pioneering initiative has been launched by the European network of Agencies for HTA (EUnetHTA; http://www.eunethta.eu/) as part of the Joint Action 3 funded by the EU. EUnetHTA has included a work package (WP5) that based on previous experience in Joint Action 2 (http://www.eunethta.eu/activities/EUnetHTA_Joint_Action_2_(2012-15)/eunethta-joint-action-2-2012–2015) promotes early-dialogue with industry and regulators on drugs and medical devices. This initiative tries to avoid further evidence generation requirements on safety and efficacy and choice of comparator when informing decision making on reimbursement. This is especially crucial when talking about SMEs that need to be efficient on evidence requirements and trials' design and are unable to afford further investments to answer HTA bodies and health care systems.

## Investment

Health care providers and organizations need to decide on which services and technologies will be implemented into the Health Systems, and, simultaneously, to which extent those goods will be funded. In view of the scarce resources and the open-ended needs that put at risk Health Systems' sustainability, the investment in a concrete condition or pathology impedes the investment in other pathologies or processes, in which similar or higher value could be generated (cost-opportunity). Unluckily, health care providers invest in health technologies that are not tailored to their needs or the settings in which they are going to be applied. Likewise, the relative added value of some health technologies at the purchasing price does not justify their acquisition and generate tensions between health care providers, health technology suppliers, and patients. More than desirable, investments are not performed on the basis of priority needs or they do not consider systems and settings characteristics acquiring technologies that are more complex than required or unsuited to the context where they will be provided, demanding expensive maintenance, or very advanced capacity building to gain the benefit of the single technology. Additionally, the variability in practice, the irrational or inadequate use of health technologies, the wide range of health professionals, and the lack of required competencies to achieve the desired outcomes may also lead to an inefficient use of investments, a spendthrift of services, and the lack of funds vital for purchasing other more priority resources for Health Systems. HTA could be helpful at this stage by anticipating the possible impact and requirements of health technologies included in different health care systems, what it has been called “Early Awareness and Alert Systems” (Packer et al., 2012) and by promoting evidence generation post-marketing authorization and reimbursement decision (Varela-Lema et al., 2012). As previously mentioned, the value of health technologies in routine care differs from that promised in theory and according to trials in ideal conditions. That's why HTA bodies, regulators, payers, health professionals, and patients are claiming for the establishment of mechanisms that headed to the generation of evidence in real practice what has been called “real world data” (Schneeweiss et al., 2016). Real world data generation supports other actions such as: “Manage Entry Agreements” (Klemp et al., 2011), adaptive licensing for innovations market authorization (Schneeweiss et al., 2016), and public procurement and prices negotiation, based on comparative effectiveness, when demonstrated value. Even legal basis had changed so that reimbursement bodies are able to spend money into the development of real life evidence (Germany—GKV-VSG, 2015).

## Disinvestment

The linear concept of health technologies life cycle was a stop to the analysis of what happened in real practice. It seemed to be that once decisions on reimbursement were taken, health technologies remained unassessed up to their disuse by health professionals. Actually, variability in practice has been a constant in health care systems. This variability could be due to the personalization of the management of individual patients or to the use of obsolete, superseded, or low-added value practices. The analysis of variability and the identification of low-added or no added-value practices has become a must. Different programs have tried to systematically identify obsolete technologies and promote disinvestment (Ibargoyen-Roteta et al., 2009; Leggett et al., 2012; Polisena et al., 2013). New initiatives like Choosing Wisely (USA http://www.choosingwisely.org/; Australia http://www.choosingwisely.org.au/home; Canada http://www.choosingwiselycanada.org/; UK http://www.choosingwisely.co.uk/; Spain http://www.msc.es/organizacion/sns/planCalidadSNS/cal_sscc.htm) promoted by systems, patients, and professional societies have achieved further attention in mass media. Notwithstanding, there is a need for further research on sources for the identification of obsolete technologies and their consequences in health care systems. The systematic, comprehensive, and accountable divestment of low added or no added value health technologies could be the basis for budget release that enables investment in innovations that seek greater value and better allocation of resources.

Future challenges are countless: from theoretical efficacy to effectiveness, from traditional “one size fits all” medicine to personalized medicine. To this evolving scenario, it must be added the limited financial resources that will force more than ever to select the best technology according to each concrete system and appropriate criteria. However, this will require stakeholders' involvement aimed at increasing quality and value of care (Stelfox et al., 2015).

## Author contributions

IG design the concept of the paper and distributed the tasks among HD and MC. HD and MC contributed to the discussion and the review of two subsequent drafts. HD contributed to the area of investment and disinvestment and MC to the discussion and drafting of the area of reimbursement and innovation. Final submission was approved by the three authors.

### Conflict of interest statement

The authors declare that the research was conducted in the absence of any commercial or financial relationships that could be construed as a potential conflict of interest.
